# Consumption

**Published:** 1882-02

**Authors:** 


### CONSUMPTION.

From official sources for the same year, it is found that of every hundred persons dying from all causes, there died in round numbers, of consumption, in

| | Consumption. | Of all lung diseases |
|---|---|---|
| Mexico City, | 3 | 17 |
| New Orleans, | 9 | 14 |
| Norfolk, | 11 | 13 |
| Philadelphia, | 15 | 29 |
| Boston, | 15 | 24 |
| New York, | 18 | 28 |
| Baltimore, | 18 | 23 |
| Charleston, | 18 | 23 |
| Havana, | 20 | 25 |

### WHERE SHALL I BUILD?

Upon the wise decision of this important inquiry depends to a greater or less extent, the health, the consequent happiness and eventual success in life, of every young farmer. It has been the experience of tens of thousands who began life hopefully, and who went to work with willing and brave hearts to "clear" a farm and make it a home for life for themselves and families, that they did well until sickness came, under which their strength and energy wilted away like a flower without water; they fell behindhand, lost their energy, ran in debt and finally settled down in the poor ambition of only meeting their expenses from month to month; their idea of getting ahead having been abandoned forever.

It is demonstrably true, that the difference of a few hundred yards, of a dozen rods sometimes, in locating a dwelling for a family, is precisely the difference between its extinction, in a few years, by disease, and its prosperity, its health, and a large family of industrious manly sons, and of refined, educated, and notable daughters. A citizen of New-York purchased a beautiful building site for a country residence, and after spending two years and a large amount of money in preparing it for the reception of his wife, children, and servants, he moved into it. Every body was delighted with the "prospect" which it afforded, of river and field and woodlands and distant mountains. With autumn came chills and fevers among his servants. He abandoned it, and never occupied it afterward, being wholly unwilling that his family should live where such a disease was possible.

A publishing house in this city erected a private residence in the country, at an expense of over thirty thousand dollars; it could be seen for many miles around; while its spacious piazzas afforded near and distant views, which delighted every visitor. During the very first year such a deadly pestilence broke out among the inmates, that it was at once abandoned, and was eventually "sold for a song." It is now known by residents on the banks of the Hudson as "Blank's Folly."

A wealthy and retired citizen of New-York built for himself a splendid mansion up-town, about four years ago, anticipating that it would be his home for life. He had occupied it but a short time, when one by one of the members of his family were taken sick. A strict examination discovered the fact that the house had been erected over a "filling," the emanations from which constantly ascending, impregnated every room in the building with deleterious gases; it was at once abandoned for another home.

The hospitals and barracks in and near Bengal are now almost useless, having been built in a locality utterly unfitted for human habitations, as far as health was concerned; their erection cost the British government sixty-five millions of dollars. This great waste of money might have been altogether avoided by the application of a very limited knowledge of the causes of disease.

From official papers presented to the British government, it is shown that of each hundred British soldiers in India, ninety-four disappear from the ranks before the age of thirty-five years, when, from military returns, it is known that "the average standard for health for Europeans in India, would compare with that existing anywhere else in the civilized world, if the known sources of disease were dried up." It is admitted, that in forty years, one hundred thousand men might have been saved, "*if proper localities had been chosen for their dwellings!*"

During the official investigations as to the causes of so much sickness and death at the National Hotel in Washington City some years ago, it was shown that there was no unusual sickness in any of the houses across the street, and that the causes of the disease were under the building itself. The symptoms in some persons were so malignant and virulent, it became the general conviction that they were the result of poison having been designedly introduced into the food. This proves the truth of the assertion already made, that the difference of a few feet in the locality of two buildings, is the difference sometimes between life and death. These things being so, it is a matter of personal happiness and pecuniary interest to every farmer who contemplates building a house, which is to be a home for himself and family, probably as long as he lives, to possess himself of such information as to enable him to ascertain certainly, why are certain localities so prejudicial to the health of families residing therein? or, in other words, what is the agent which causes disease in this mysterious manner? It may seem discouraging at first view to state that this destructive agency is as invisible as the viewless wind; at the same time it will afford encouragement to be assured that its nature is known, as also some of the laws by which it is regulated; and that by an easy attention to them, the Samson may be shorn of his locks; and the great destroyer may either be avoided, or rendered as harmless as the gentlest touch of infancy.

The name of this perfectly remorseless destroyer of human life is

"MIASM,"

From a Greek word which means *emanation;* that is, ARISING FROM; because it comes up from the surface of the earth. It is a short word, but it brings weary sickness and agonizing death to hundreds of thousands every year; it will bring sickness and death, sooner or later, to many a reader of this article; but a sickness and death which could have been avoided.

MIASM is the principal cause of nearly every "epidemic" disease; that is, of every sickness which "falls upon the people;" attacking numbers in any community, such as fever and ague, diarrhea, dysentery, cholera, bilious, intermittent, congestive and yellow fevers. But it is gratifying to know that it is an avoidable cause of disease. Money and wisely directed efforts can banish it from almost any locality. All that is needed is to know the laws of miasm, and wisely adapt ourselves to them.

In 1860, one of the daily papers of New-Orleans stated: "The yellow fever has broken out in the city under every conceivable variety of circumstances; when the streets were clean and when they were filthy; when the river was high and when it was low; after a prolonged drought, and in the midst of daily torrents; when the heat was excessive, and when the air was spring-like and pleasant; when excavations and disturbances of the soil had been frequent, and when scarcely a pavement had been laid or a building erected. Almost the only fixed and undeniable fact connected with the disease is, that its prevalence is simultaneous with the heats of summer, and that frost is its deadly enemy." Here, then are two important laws of miasm; and scientific observation directed to that special point in all countries, confirms the two great truths, that,

First. Miasm prevails in hot weather.

Second. Miasm can not exist as a cause of disease in cold weather.

Third. An inference is drawn embodying a third law of miasm, which is, that it is a cause of disease only from June to October, in our latitudes.

Fourth. A fourth law of miasm is confirmed by the now historical fact, that for three summers yellow fever was not known as an epidemic in New-Orleans; because, from the scientific views held by those in power in that city in the early summer of 1861.

It is within the memory of the present generation, that some thirty years ago or more, the city of Louisville, in Kentucky, was one of the most pestilential spots in the habitable West. But by a wise system of filling and draining, it is now one of the healthiest, as well as one of the most beautiful cities of the great valley.

We have then arrived at four controlling facts in reference to miasm—that heat and moisture are essential to its production in any locality; that it can not exist where there is severe frost or great dryness.

But as it is known the world over that miasm never exists in deserts, where there is nothing but dry sand and a burning heat, it is clear that something more than heat is necessary to cause miasm.

bushes, or hedges or trees, between a miasm-producing locality and a dwelling, antagonize the miasmatic influences; the living leaves seeming to absorb and feed upon the miasm; but there should be a space of fifty yards at least between the hedge and the house; and the thicker and broader and higher the hedge, the better; and the nearer the leaves are to the ground the better, for the miasm gropes on the surface in its greatest malignity: and is seldom concentrated enough at the hight ot ten feet to be materially hurtful to man, unless it comes up a slope Hence, in the old cities of the world, in the times of plagues and pestilences, the people who could not "go to the country," had a custom among them to live in the upper stories of their dwellings, while the sickness raged; they would not even come down stairs to obtain marketing, but would let down baskets by ropes to the country people, for the provisions they had to sell. But they failed to discover why the country people could come to town with impunity, while they themselves were safe from disease in proportion as they lived in the upper stories of their dwellings. But a law of miasm has since been determined which beautifully unravels the mystery. Miasm is condensed by cold, made heavy, and falls to the earth, hovering, as it were, within a foot of its surface; hence is not breathed, unless a man sleeps on the ground. On the other hand, heat so rarefies miasm, as to make it comparatively innocuous. Hence the coolness of the early morning and of sundown threw the miasm to the surface, by condensing or concentrating it, and thus making it heavy; while the heat of the day, of a summer's sun, so rarefied and lightened the miasm, as to send it upward to the clouds. *The country people came to town in the daytime!*

Less than fifty years ago, the yellow fever and other deadly diseases prevailed in Charleston, South-Carolina, and it was known to be certain death, except to the very hardy or the acclimated, to sleep in the city a single night. Yet the merchants came to town at mid-day, under a blistering July sun, with perfect impunity. Hence, from June to October, it is best for farmers' families to sleep in the upper stories of their dwellings. In this connection, it is practically useful to know that the most malignant agencies of nature may be rendered harmless of miasm. A neglect of such a simple precaution, in certain districts where malaria is known to exist in a concentrated form, is a cause of death so common as to be known and guarded against by the most uneducated laborers. A gentleman, a native of the city of Rome, informed the writer that multitudes of agricultural laborers who have been employed during the day in the low, level damp fields near the city, come into town about sundown and sleep in the streets and on the steps and stoops of houses, in order to avoid the sickly atmosphere of the evening in the "marches." No less a personage than a young king lost his life within two years, under the following circumstances: Having to pass the night in one of his journeys at a house located in the midst of an extensive low land or marsh, and wishing to be on horseback early in the morning for a hunt, the landlord pressed upon him the danger of being out early, and that at least he should take his breakfast first. The impatient youth was observed early next morning sitting at his open window, enjoying, as he thought, the delightful air as it blew in upon him, and soon after ordered his horses. He became ill and died of fever in a few days. The writer has lived among the Creoles of Louisiana where vegetation is rank in swamps, upon which the hot summer's sun beams with fiery power for many hours every day; but they are proverbially exempt from fevers, as are Northerners also, who adopt the habits of the Creoles—that is, to have their breakfast, or at least a cup of hot strong coffee with milk, brought to their bedsides *before they get up* of a morning. The value of this practice is known and appreciated all over the South; so that while it is greatly better to locate a house where miasm can not reach it from ponds or sluggish streams or bottom lands, a farmer whose house is already thus situated, is not without an efficient remedy in the plan proposed above.

But there is another infallible remedy against miasmatic diseases as to families who feel themselves compelled to live in a house exposed to miasm. It was stated awhile ago that heat so rarefied miasm as to render it innocuous. No family can be troubled with fever and ague in any ordinary locality, if from June to October a brisk fire is kindled in the family-room, to turn for an hour about sunrise and sunset, and if the family are required to repair to that room morning and evening and remain there, at least until they get their breakfast in the morning, and their supper at the close of the day.

It follows then that ordinarily, there is nothing unhealthful in the night-air after supper; on the contrary, health would be promoted, and important social benefits would accrue to country neighborhoods, if two or three nights of every week, after tea, were spent in friendly visiting, remaining not later than ten; thus encouraging that interchange of social associations which diffuses intelligence, promotes kindly feeling, enlarges the views, expands the ideas, and elevates the whole character, by cultivating the tastes as to dress, tidiness of person, and the imitation or copying after any ornament or improvement of the grounds and dwellings of the neighborhood. In this way, one intelligent practical farmer in a neighborhood, by occupying a house which he has built or remodeled for himself, so as to have all the comforts and conveniences which knowledge and observation and experiment have found to contribute largely to the health, happiness, and thrift of the occupants, will prove a leaven which shall spread from one habitation to another in a comparatively short time, until every dwelling in the circuit of many miles will be more or less improved, and thus the face of the whole country be changed for the better, with the promise and realization of a further progress, onward and upward.

### RECAPITULATION.

Although the statements which have been made were presented in connection with the selection of the most healthful locality for building a new family residence, they are practically applicable to all cases wherein it may be desirable to make a house already built more comfortable and more healthful than it is, because, from what has been stated, it will be seen that a dwelling already erected should not be hastily and blindly abandoned merely on account of its insalubrity, for in the light of the above statements, it may be found that the causes of any present sickness are of a transient or of a remediable character, which may thus be illustrated:

The most favorable circumstances for the production of a miasmatic epidemic, speedy, malignant, and wide-spreading, are the exposure of the muddy bottom of a pond or sluggish stream to the beaming heat of a summer's sun. In less than a week whole neighborhoods have been stricken with disease, yet under such circumstances, and according to the well-established laws of miasm, five families may dwell within half a mile of a drained mill-pond, and yet only one will suffer from it, while the other four will remain exempt from unusual disease:

First. If a rapid stream half a mile wide runs between the drained pond and the house.

Second. If there is interposed a thick hedge or growth of living luxuriant trees or bushes. A treble row of sun-flowers are known to have answered the purpose in repeated cases.

Third. If the prevailing winds from June to October are from the house toward the pond.

Fourth. If the house be on a steep hill.

The reasons for the above exemptions are here shortly recapitulated:

First. Miasm does not cross a wide, rapid stream.

Second. Miasm is absorbed by thick, living luxuriant foliage.

Third. Miasm can not travel against the wind.

Fourth. Miasm can not ascend a high, steep hill.

There is no mystery in these variations, nor any complexity, when the laws of miasm are thoroughly understood.

It will be practically useful for the young farmer, in a pecuniary point of view, to understand further that in one year a house on the banks of a mill-pond or sluggish stream may be visited with sickness; the very next year that same house may be exempt, because it is a very cold summer; the third year it will escape, because it is a very hot summer; the fourth year it will be a very healthful habitation, because it has been a very wet summer. Why these variations?

First. Miasm can not form, or if it does, can not rise through a foot or two of depth of water, and the wet summer kept the bed of the pond covered.

Second. The hot summer dried the bed of the pond to dust, and there can be no miasm without dampness.

Third. The cold summer did not give the degree of heat necessary to the generation of miasm—that is, eighty degrees of Fahrenheit.

These principles fully explain the apparent mystery of the epidemics in New-Orleans, already referred to in the first part of this paper.

An illustration of the laws of miasm, which the reader will never forget, was had during a cholera summer in Boston, under the following circumstances: the city authorities inaugurated a most perfect system of cleanliness. Efforts were made to procure the services of the most reliable men to visit every house from cellar to garret, and compel the removal of every thing which could have even a remote tendency to invite the fearful scourge. The results were admirable; there was not a single case of cholera except in a very restricted district—in fact one family only was attacked. A more special examination was instituted, when there was found in a remote corner of the cellar a large pile of the accumulations of bad housekeeping for years; and this was in a state of putridity. On its removal, and the plentiful use of the most powerful disinfectants, the disease at once disappeared and did not return!

### CELLARS IN DWELLING-HOUSES.

With a fact like the above staring one in the face, and in connection with another, that farmers generally make their cellars the winter and summer receptacles of every variety of vegetables and fruits, more or less of which are put away in a bruised, rotted, or unripe condition, and thus speedily become putrid, by acetous fermentation without the aid of much heat, it is apparent that these gases are constantly ascending, and must unavoidably impregnate every room in the house with a vitiated and unwholesome atmosphere; and in consequence of another known fact, and unfortunately almost universal, that the cellar being convenient and "out of sight" of visitors, is made the receptacle of all that is old and unseemly as well as of kitchen offal, by the laziness of bad housekeepers or unprincipled servants. For these considerations, it is clear that no cellar should be built under that part of a house which is to be occupied as a place to eat and sleep and live in, whether in town or country. The great cost of land in towns and cities may be some apology for having cellars underneath; but there is none for having cellars under a farm-house. Where there are already cellars, a great deal may be done toward preventing them from becoming the fruitful source of sickness and suffering; and if there is any obscure or slow disease in the family of any reader of this article, and a cellar is attached to the building, it is worth the experiment to secure the following "alterations" as to the cellar: let the cellar be emptied of every movable thing; let the walls and floor be thoroughly swept, and, if practicable, washed; and after being allowed to "air" for a week or two, have the ceiling plastered, the space between that and the floor having been filled with dry sand or gravel, or ashes or pulverized charcoal, not only to keep dampness and cold and any hurtful chance emanations coming up from below, but also, in case water should leak through the floors, it might easily pass through the sand, and thus perfect dryness be secured. The walls should be smoothly plastered, and the floor covered with a hard cement, thick, smooth, and strong; and both walls and ceiling should be well whitewashed twice a year; once a year, at least, the old whitewash should be scraped or swept off, before the new is applied. The best, because the cheapest and most universally available whitewash, is made as follows: put unslaked lime, that which is in the form of the original rock, in a vessel; pour boiling water on it until it is covered; place a cloth over the vessel so as to confine the most minute particles of the lime, they being the ones which most perfectly "penetrate" the surfaces to which the wash is applied, and consequently remain the longest. Subsequently dilute the wash to the consistence of thick cream, and apply it thoroughly and thickly, thus accomplishing two objects, a white, light-giving surface, having a "body," as painters term it, which is capable of absorbing, and thus rendering harmless the "bad" airs or gases which may be formed in the cellar.

Every partition and every shelf in a cellar should be made of smoothly planed boards, well covered with good white paint, thus preventing the accumulation of dust, and aiding in making the cellar light, cheerful, and clean, for the more light you can have, the better. Every cellar should be so contrived, that either by its grating or windows or doors, it may be easily and thoroughly ventilated, an hour or two at least every day in the year

It is scarcely necessary to remark, that if a cellar is liable at any time of the year, even for a few days, to have water rise and stand on the floor or even to have the floor a little wet, draining tiles should be put under it before the floor is "cemented." All shelves in a cellar should be so arranged that you can go all around them; it is not advisable to put any shelving against a cellar-wall; and if all the shelves are suspended from the ceiling, so much the better on several accounts, not the least of which is that more "floor-room" is thus obtained.

When a house is to be erected in a new locality, and it has been wisely determined to have the cellar off from the family building, but yet to be easily accessible from the kitchen without having to go "out of doors"—say under the kitchen itself or under the wood-house, or simply under the ground, its roof being a part of the front yard or garden, if you please, but so covered over with soil and grass, bushes, etc., that it would not be known to be there—the next point is to arrange that the foundation of the house should be on a rock, at least three feet deep, and on a spot descending, if possible, in every direction. The walls of the house should be at least two feet above the surface of the earth, crevices having been left at intervals on each side, so as to admit a free circulation of air, but not large enough to admit mice. There should be an open ditch all around the inside of the wall, as a drain to any dampness, with a sufficient descent, at least at one point, to insure the drain to be passed off.

It is well to plaster a foundation wall inside and out, and to have every stone well laid in a good mortar, not being sparing of lime or sand in its preparation. Too much "loam," or common dirt, is generally used, so that the mortar crumbles to powder, has no tenacity, no binding power, instead of hardening and becoming a part of the wall itself.

The space between the lower edge of the joists of the ground floor and the upper edge should be filled with dry sand, ashes, or, which is much better, charcoal, for the three-fold object of, first, keeping the lower floor dry; second, keeping it warmer in winter; third, absorbing any deleterious gases which might arise from the ground. As to the materials for building, each locality has its peculiar conveniences, but it should not be forgotten that wooden buildings are best for the country, because they are dryer, and consequently more healthful.

The best kind of roof for a country house is the old-fashioned steep roofs, with a "comb" in the center; with no "hips" or dormer windows; these may make a building more picturesque, but they so generally leak, that a plain, steep, shingled roof is safer, more economical, and more universally available.

As to the shape and size and hight of the rooms, each builder must decide for himself, according to his taste and the length of his purse. A square building gives most room for the same money; and a broad hall in the center of the building affords greater advantages than any other arrangement.

"High ceilings," as they are called, are now much the fashion; but they are more costly in the first place, and occasion an unnecessary waste of fuel ever thereafter; they are commended for their spaciousness; but they sometimes give a barn-like appearance to a house, and are never so cosy as rooms which are not quite so high.

Winding stairs are objectionable everywhere, but especially in the country, where persons rise by daylight or sooner, and where there are old persons or young children; as in haste or darkness there is danger of falling, and breaking or disjointing the limbs or neck.

It is a great saving in the cost of furniture, if, in the erection of new buildings, and in the modification of old ones, large, light, and roomy closets are plentifully supplied, and with them shelves, hooks, and drawers. Many persons in the country when "dressed," show bad housekeeping and characteristic slovenliness, by having their outer garments marked with innumerable "creases," showing that they have been thrown negligently into a drawer, and allowed thus to remain from one "going-out" to another. The outer dresses of both sexes should be hung up in closets, protected by doors from dust; and to this end, every farm-house should have a great abundance of closet-room. These closets should be always large, and all the doors should be hinged within two or three inches of the wall, so that there may be no dark corners for the collection of dust or other improper thing, or for the hiding of what is valuable.

